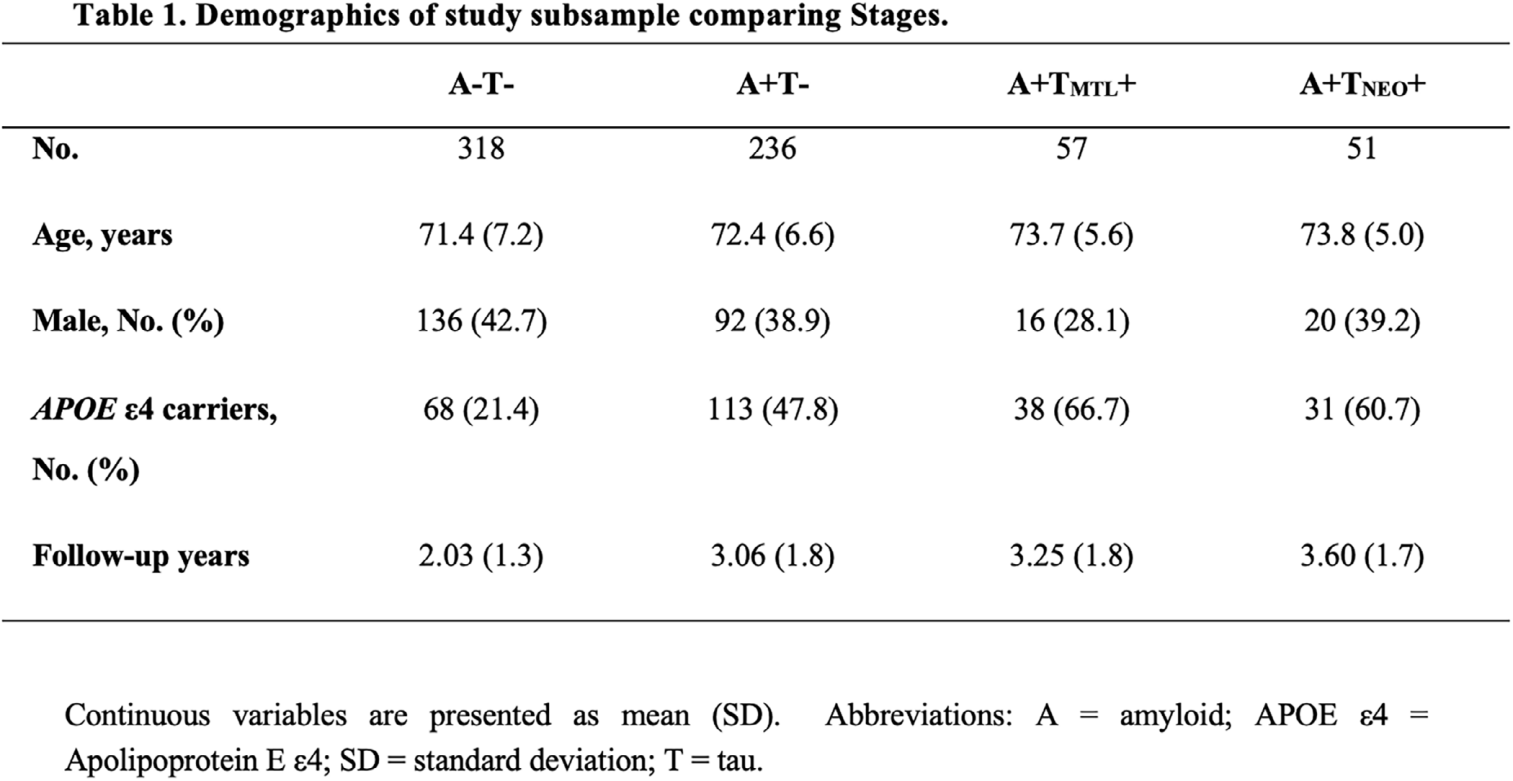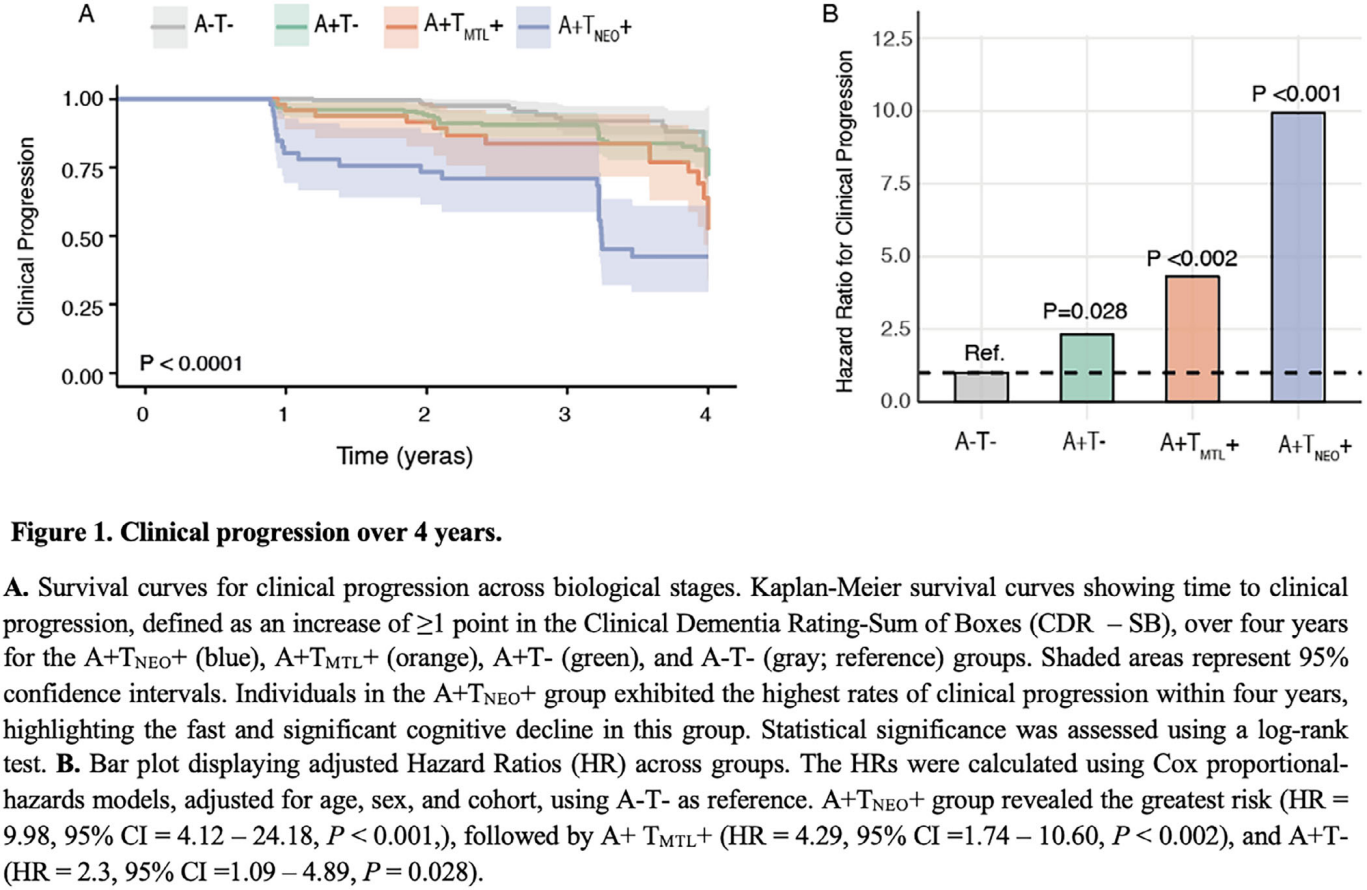# Imaging‐based Biological Staging for Alzheimer's Disease Prognosis

**DOI:** 10.1002/alz70856_107452

**Published:** 2026-01-09

**Authors:** Marco Antônio Albini Valer, João Pedro Ferrari‐Souza, Lorenzo Fontura Brasil Barcellos, Isabela Just de Jesus Vanni, Andrei Bieger, Douglas Teixeira Leffa, Firoza Z Lussier, Wagner S. Brum, Cristiano Aguzzoli, Anderson Corin, Marco De Bastiani, Giovanna Carello‐Collar, Wyllians Vendramini Borelli, Nesrine Rahmouni, Joseph Therriault, Lydia Trudel, Arthur C. Macedo, Pamela C.L. Ferreira, Guilherme Povala, Bruna Bellaver, Diogo O. Souza, Pedro Rosa‐Neto, Tharick A Pascoal, Eduardo R. Zimmer

**Affiliations:** ^1^ Pontifícia Universidade Católica do Rio Grande do Sul, Porto Alegre, Rio Grande do Sul, Brazil; ^2^ University of Pittsburgh, Pittsburgh, PA, USA; ^3^ Universidade Federal do Rio Grande do Sul, Porto Alegre, Rio Grande do Sul, Brazil; ^4^ McGill University, Montreal, QC, Canada; ^5^ Neurology Department, São Lucas Hospital of PUCRS, Porto Alegre, Rio Grande do Sul, Brazil; ^6^ Universidade Federal de Pelotas, Pelotas, Rio Grande do Sul, Brazil; ^7^ Universidade Federal do Rio Grande do Sul, Porto Alegre, RS, Brazil; ^8^ Universidade Federal do Rio Grande do Sul, Porto Alegre, Brazil

## Abstract

**Background:**

Recent revised criteria for Alzheimer's disease (AD) emphasize the potential utility of imaging biomarkers in disease staging. Although promising, the practical applicability of staging schemes requires further investigation. In this study, amyloid‐β (Aβ) and tau positron emission tomography (PET) were used to evaluate the prognostic performance of the imaging‐based biological staging criteria in cognitively unimpaired (CU) individuals.

**Method:**

This longitudinal analysis involved 662 CU individuals: 467 participants from the Alzheimer's Disease Neuroimaging Initiative (ADNI) cohort and 195 individuals from the Anti‐Amyloid Treatment in Asymptomatic AD (A4) study placebo group. In the ADNI cohort, Aβ positivity (A+) was defined as standardized uptake value ratios (SUVR) >1.11 for [18F]Florbetapir and >1.08 for [18F]Florbetaben. In A4, Aβ positivity was established via visual reading. Tau tangles were assessed using [18F]Flortaucipir for both cohorts, with positivity defined as 2.0 standard deviations (SD) above the mean SUVR of Aβ‐negative CU reference groups (ADNI:*n* = 352; LEARN A4 substudy: *n* = 55). These tau PET cutoffs, derived from the medial temporal lobe (MTL) and neocortex (NEO) regions, were applied to classify individuals as T_MTL_+ and T_NEO_+. We used Kaplan‐Meier curves and Cox proportional hazard models to evaluate the 4‐year risk of clinical progression (i.e., one point increase in the Clinical Dementia Rating ‐ Sum of Boxes) according to imaging‐based baseline groups.

**Result:**

The mean (SD) age of the study population was 72.1 (6.8) years, and 264 (39.9%) were men (Table 1). Kaplan‐Meier curves demonstrated that the A+T_NEO_+ group presented a separate survival probability curve compared to the other groups (Figure 1A). Cox proportional‐hazards models corroborated the greatest risk of clinical progression in the A+T_NEO_+ (HR = 9.98) group, followed by the A+T_MTL_+ (HR = 4.29) and A+T‐ (HR = 2.31) groups, in comparison to the reference group (A‐T‐; Figure 1B).

**Conclusion:**

The integration of Aβ and tau PET imaging provides critical prognostic information in early disease stages, with neocortical tau deposition performing as a robust predictor of clinical progression. These findings reinforce the utility of imaging biomarkers for AD staging and prognosis, highlighting that the topography of tau tangle accumulation is a key factor in enhancing risk stratification of individuals with preclinical AD.